# Multidrug resistant 1 (MDR1) C3435T and G2677T gene polymorphism: impact on the risk of acute rejection in pediatric kidney transplant recipients

**DOI:** 10.1186/s13052-023-01469-w

**Published:** 2023-05-18

**Authors:** Mai S Korkor, Tarek el-desoky, Youssef M Mosaad, Doaa M. Salah, Ayman Hammad

**Affiliations:** 1 Pediatric Nephrology Unit, Mansoura University Children’s Hospital, Faculty of Medicine, Mansoura University, Mansoura, Egypt; 2 Pediatric respiratory and allergy Unit, Faculty of Medicine, Mansoura University Children’s Hospital, Mansoura University, Mansoura, Egypt; 3 Clinical Immunology Unit, clinical pathology department and Mansoura Research center for cord stem cells (MARC_CSC), Faculty of medicine, Mansura University, Mansoura, Egypt; 4 Pediatric Department, Pediatric Nephrology Unit & Kidney Transplantation Unit, Cairo University Children Hospital, Kasr Al-Ainy Faculty of Medicine, Cairo University , Cairo, Egypt

**Keywords:** Multidrug resistant 1 gene, Acute rejection, Tacrolimus pharmacokinetics, Kidney transplantation

## Abstract

**Background:**

Tacrolimus is the backbone drug in kidney transplantation. Single nucleotide polymorphism of Multidrug resistant 1 gene can affect tacrolimus metabolism consequently it can affect tacrolimus trough level and incidence of acute rejection. The aim of this study is to investigate the impact of Multidrug resistant 1 gene, C3435T and G2677T Single nucleotide polymorphisms on tacrolimus pharmacokinetics and on the risk of acute rejection in pediatric kidney transplant recipients.

**Methods:**

Typing of Multidrug resistant 1 gene, C3435T and G2677T gene polymorphism was done using polymerase chain reaction-restriction fragment length polymorphism (PCR-RFLP) for 83 pediatric kidney transplant recipients and 80 matched healthy controls.

**Results:**

In Multidrug resistant 1 gene (C3435T), CC, CT genotypes and C allele were significantly associated with risk of acute rejection when compared to none acute rejection group (P = 0.008, 0.001 and 0.01 respectively). The required tacrolimus doses to achieve trough level were significantly higher among CC than CT than TT genotypes through the 1st 6 months after kidney transplantation. While, in Multidrug resistant 1 gene (G2677T), GT, TT genotypes and T allele were associated with acute rejection when compared to none acute rejection (P = 0.023, 0.033 and 0.028 respectively). The required tacrolimus doses to achieve trough level were significantly higher among TT than GT than GG genotypes through the 1st 6 months after kidney transplantation.

**Conclusion:**

The C allele, CC and CT genotypes of Multidrug resistant 1 gene (C3435T) and the T allele, GT and TT genotypes of Multidrug resistant 1 gene (G2677T) gene polymorphism may be risk factors for acute rejection and this can be attributed to their effect on tacrolimus pharmacokinetics. Tacrolimus therapy may be tailored according to the recipient genotype for better outcome.

**Supplementary Information:**

The online version contains supplementary material available at 10.1186/s13052-023-01469-w.

## Background

Kidney transplantation (KT) is the optimal treatment option for children with end stage kidney disease (ESKD) [1]. It provides them with better survival, growth and quality of life compared to those remaining on dialysis [2].

Despite of advancement in the field of KT; the long-term graft outcome is not yet favorable in pediatric population, due to associated complications as recurrent infections, acute rejection (AR), poor adherence to immunosuppressive (IS) drugs and transplant glomerulopathy (TG) [1, 3].

The incidence of AR has decreased with availability of potent IS drugs, but it is still a major risk of early graft dysfunction and late allograft loss [4]. AR can occur at any time after KT but commonly in early post-operative months with declining incidence thereafter [5]. Allograft biopsy is the golden standard for diagnosis of AR. It can be classified according to the Banff pathological criteria into T cell mediated rejection (TCMR), antibody mediated rejection [6] and mixed rejection [7].

Tacrolimus (TAC) is the cornerstone of most IS regimens with a characteristic narrow therapeutic window [8]. Although many factors, including age, ethnicity, and organ function can influence the drug effects, the pharmacogenetics play a critical role in interindividual variability in drug disposition and effects [9]. TAC is a substrate for multidrug resistant 1 (*MDR-1*) gene which is also referred to as ATP Binding Cassette (*ABC*) transporters, located on chromosome 7q21, comprises 28 exons and encodes for Permeation glycoprotein (P-gp). P-gp acts as a membrane efflux pump transporting several molecules through the cell membrane of various epithelial, endothelial cells and lymphocytes [10].

Single nucleotide polymorphisms (SNPs) are the most frequently inherited genetic variations among people that occur frequently, every 100–300 bp (bp) [11]. Several SNPs have been reported in the MDR1 gene which can affect the metabolism of drugs, the pharmacological action and toxicity profile of a vast number of therapeutic agents [12].

The first defined mutations of MDR1 gene were G2677T/A, C3435T and G2995A [13]. C3435T, SNP is a silent mutation that is located in exon 26 and affects the expression and function of P-gp [14]. In spite of being silent mutation that does not change the coding sequence of the target protein, it can affect rate of protein translation, folding and activity. Therefore, it can eventually affect the pharmacokinetics of drug substrate of MDR1 [15]. G2677T, SNP results in substitution of Alanine amino acid by serine at position 893 of amnio acid chain of P.gp [16] while G2677A mutation substitution of Alanine amino acid by Threonine [17]. G2677T/A polymorphism can alter the expression and activity of P-gp and thus affects in vivo drug disposition and its therapeutic effects [18]. Taking into consideration that 2677 A has very low frequency [19], G2677T only was studied together with C3435T in the current study.

The associations between MDR1 genotype and the pharmacokinetics of TAC remain unclear. Although, It was concluded from some studies that significant differences in TAC trough level exist between different MDR1 genotypes [8, 20]. Nevertheless; other researchers did not find an association between genotypes and TAC trough levels [21, 22].

It has been demonstrated that, linkage disequilibrium (LD) between C3435T polymorphism in exon 26 and G2677T in exon 21 may contribute to functional alteration rather than the effect of single haplotype variation [23]. Interestingly, this LD varies between different ethnic groups [24]. In addition, the data available in children are limited and it is evident that the pharmacokinetics are different between adults and children due to variation in plasma binding proteins, altered expression of intestinal P-glycoprotein and increased 1st pass metabolism [25]. Thus, we conducted the current study to investigate the impact of MDR1, C3435T and G2677T SNPs on TAC trough levels and on the risk of developing AR in a cohort of Egyptian pediatric kidney transplant recipients (KTRs).

### Patients and methods

This is a cross sectional, case control study that included 163 participants; 83 pediatric KTRs and 80 healthy controls. Pediatric KTRs were recruited during their follow up at Kidney Transplantation Outpatient Clinic, Cairo University Children Hospital (Abo El Reech Hospital). Age and sex matched healthy controls were recruited during their routine checkup at General Pediatric Outpatient Clinic. The study was conducted over two years (from January, 2020 to December, 2021). An informed consent for enrolment in the study was obtained from the legal guardians of all participants. The protocol of the study was approved by Mansoura Faculty of Medicine Institutional Research Board **(MD.20.02.283)** and by Pediatric Nephrology Unit, Pediatric Department, Faculty of Medicine, Cairo University.

**Fig. 1:**
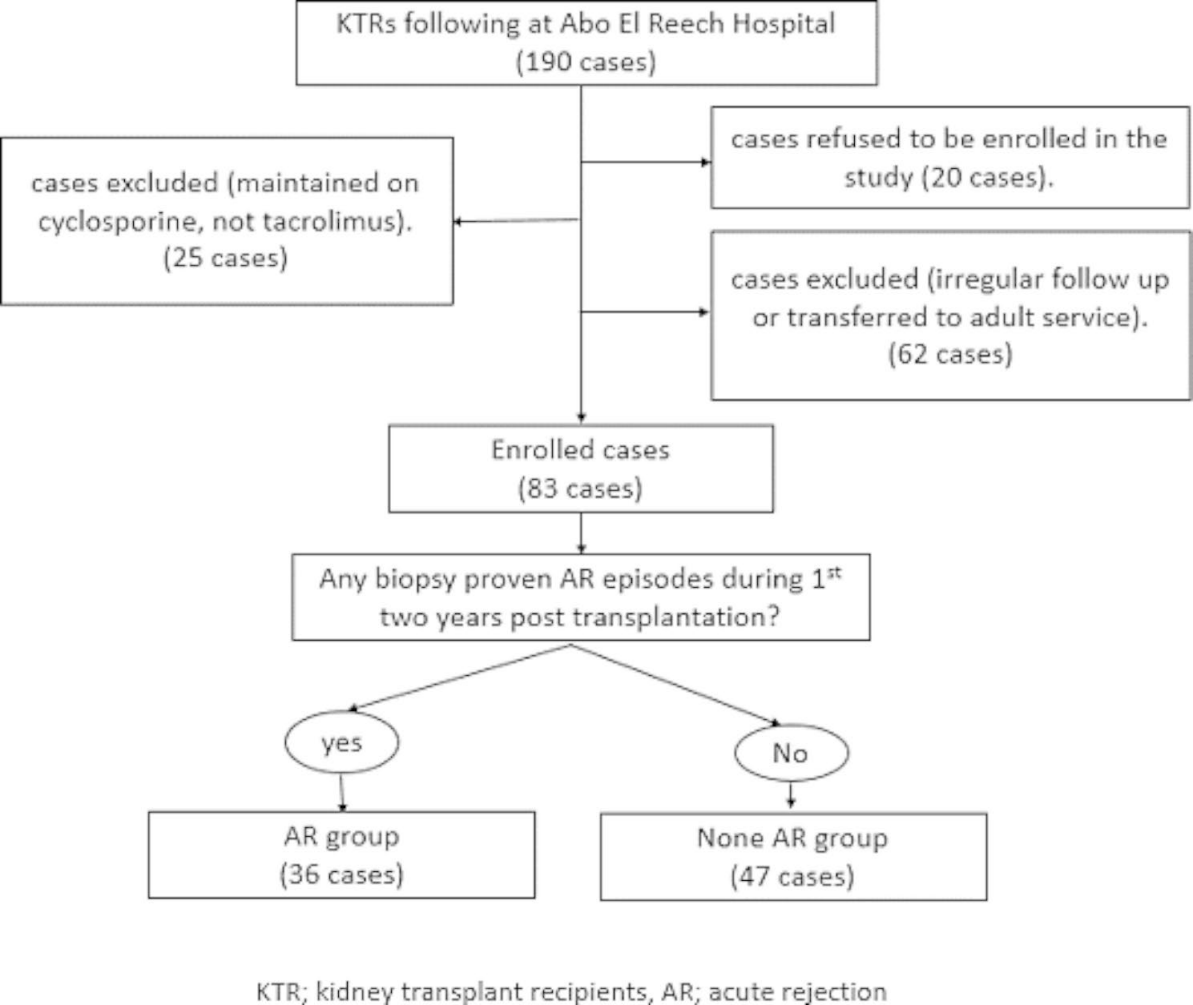
Diagram showing the flow of participants during enrollment in the study

Patients were enrolled into the study according to the following criteria: (a) recipients of living donor kidney transplant (b) aged between 2 and 18 years (c) receiving TAC as a part of their maintenance immunosuppressive protocol (d) following up for at least two years after KT. Patients who received cyclosporine as maintenance therapy, had irregular follow up visits, transferred to adult service or refused to be enrolled in the study were excluded from the study as in Fig. 1.

The included KTRs (n = 83) were divided into 2 groups: (1) AR group (n = 36): KTRs with one or more of AR episode experienced during their follow up and (2) None AR group (n = 47): KTRs with stable graft function (SGF) for at least two years after KT. AR was defined as rise in serum creatinine of 20–30% from baseline levels and confirmed by pathological evidence of immune mediated graft damage that can occur at any time posttransplant, but more commonly in early postoperative months [5]. All rejection cases were biopsy proven by allograft biopsies that were processed and analyzed by single expert pathologist in the field of kidney transplantation. SGF was defined as serum creatinine < 1 mg/dl and no decline in GFR or change in graft function within the last 6 months [26]. However, all the included cases in the none AR (SGF) had follow up duration for at least 3 years post-transplantation.

As regard the centre policy for treatment of AR, all cases started treatment with 3 IV pulses of methylprednisolone (150–250 mg/m2/dose), initiated even prior to graft biopsy and followed by rapid tapering of oral steroids to or just above the maintenance dose. Further treatment of AR was determined according to the pathological findings in allograft biopsy: a) Anti-thymocyte globulin (ATG) was given in all steroid resistant acute TCMR that was defined as no response within 5–7 days after the first dose .b) options for acute ABMR included plasma exchange (PEX), intra venous immunoglobulins (IVIG) and anti-CD20 monoclonal antibody (rituximab) [27].

### Base line, clinical and transplantation related data

Basic data were collected at enrolment in the study including: age, sex, original renal disease either congenital anomalies of kidney and urinary tract (CAKUT) or non CAKUT [28]. CAKUT included obstructive uropathy as posterior urethral valve (PUV) and pelviureteric junction obstruction (PUJO), developmental anomalies of the kidneys as aplasia, hypoplasia or dysplasia, vesicoureteric reflux (VUR), polycystic and multicystic dysplastic kidneys (MCDK), hydronephrosis, duplex kidney, duplicated collecting system and megaureter [29], While non CAKUT included cases with nephrotic syndrome (FSGS), lupus nephritis membranoproliferative glomerulonephritis (MPGN) and chronic interstitial nephritis. Data regarding need of kidney replacement therapy (KRT) and its duration, need for native nephrectomy (s), weight, height and body mass index (BMI) calculation [30] were collected. Immunological risk and CMV status prior to transplantation [31], induction and maintenance immunosuppressive therapy and TAC induced side effects were documented for all cases. For AR group, onset of AR after KT, pathological type of rejection, anti-rejection therapy and the response to it were reported.

All KTRs received antibody induction therapy (either antithymocyte globulin (ATG) or Basilximab) and were maintained on TAC based triple IS regimen together with prednisolone and mycofenolate mofetile (MMF) according to the adopted protocol [6]. Only one patient received azathioprine as adjuvant therapy instead of MMF due to intolerance to severe gastrointestinal adverse effects. None of the included cases was maintained on mammalian target of rapamycin inhibitor (mTORi).

Tacrolimus was started at a dose of 0.15 mg/kg/day in 2 divided doses and then the dose was adjusted according to the trough level measured before the next dose. The accepted trough level in our protocol is 10–12 ng/ml in 1st month and 8–10 ng/ml till 3 months, 7–8 till 6 months and 6–7 till the end of the first-year after KT [6]. Daily weight adjusted dose of TAC and its trough level during 1st 6 months after KT were recorded. Concentration/dose (C/D) ratio was calculated by dividing TAC trough blood concentration (ng/ml) by the corresponding weight adjusted daily dose (mg/kg/day).

### Single nucleotide polymorphism (SNP) genotyping

**Fig. 2:**
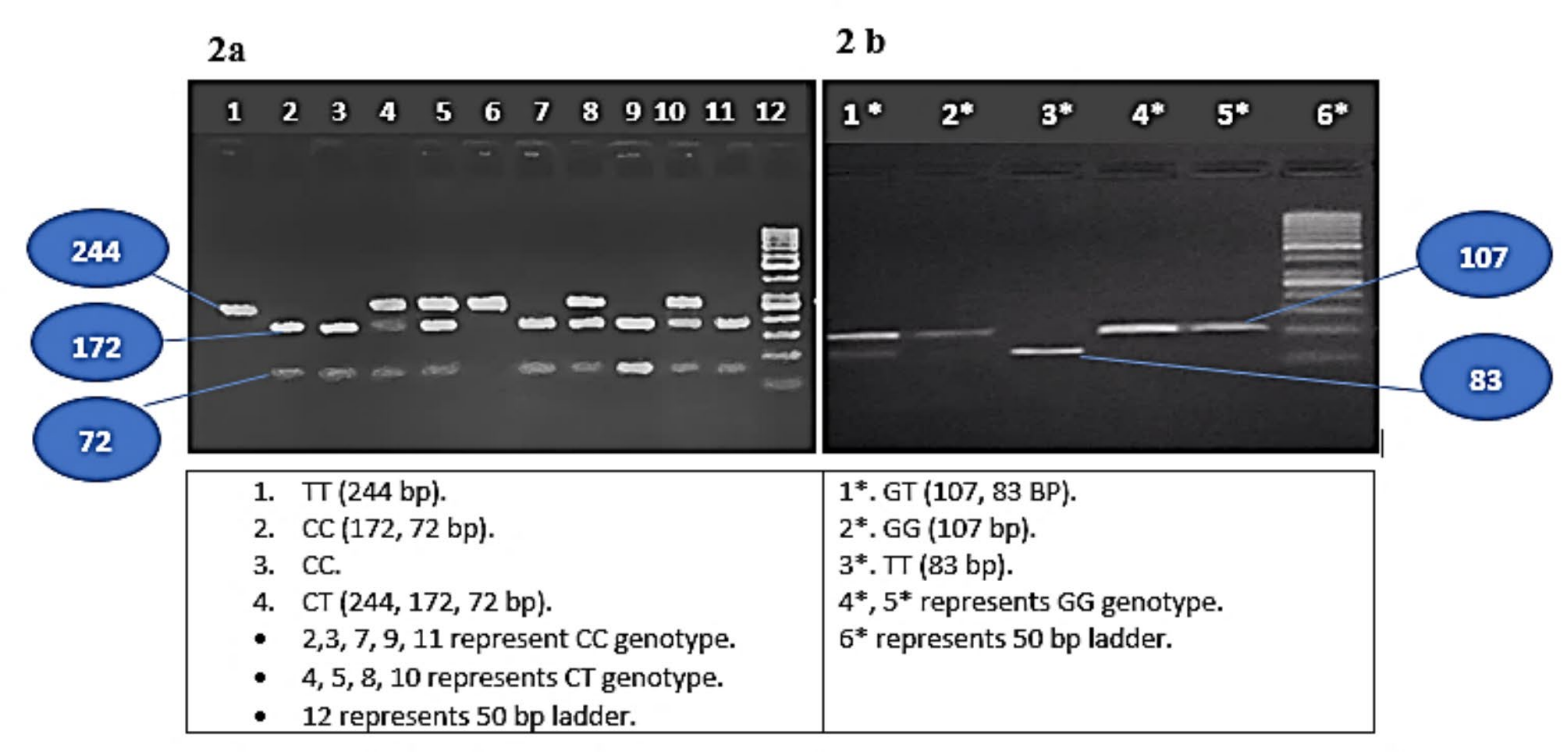
PCR products and genotypes, **2a** represents MDR1 C3435T and **2b** represents G2677T

MDR1, C3435T SNP, is composed of C and T alleles, C is the ancestral allele. [10]. MDR1, G2677T is located on exon 21, and is composed of G and T alleles, G is the ancestral allele. Both are located on chromosome 7 within ATP binding cassette subfamily B member 1 (ABCB1). Genomic DNA was extracted from whole venous EDTA blood using Thermo Scientific Gene JET whole Blood Genomic DNA Purification Mini Kits (QIAGEN, Germany) according to manufacturer’s instructions [32] and then stored at − 20°C until use. The genotypes of *MDR1* SNPs were analyzed by the polymerase chain reaction-restriction fragment length polymorphism (PCR–RFLP) method using the following primers: For C3435T, we used forward primer 5´ - GATCTGTGAACTCTT GTT TTCA − 3´ and reverse primer 5´ - GAAGAGAGACTTACATTAGGC − 3´. For G2677T, forward primer 5’-TACCCATCATTGCAATAGCAG-3’ and both 5’-TTTAGTTTGACTCACCTTGCTAG-3’ and 5’-TTTAGTTTGACTCACCTTTCTAG-3’ were used as reverse primers. [33]. Reaction volume was 25 µl: 5 µl DNA at 100 ng/µl, 15.0 µl DreamTaq Green mater mix (Fermentas, Germany), 0.5 µl of each primer (25 pmol/ µl), and 4.0 µl H2O. Reaction conditions were carried out in thermocycler PTC-100 (Biorad, USA), with the following cycling parameters. The PCR conditions included an initial 94 °C for 5 min followed by 35 cycles of 94 °C for 40 s, 60 °C for 40 s, and 72 °C for 40 s and a final extension at 72 °C for 7 min. We used restriction enzyme MboI and XbaI respectively [34]. 10 µl of PCR products were resolved in 2% agarose gel to check the PCR products. For MDR1 C3435T, bands of 172, 72 correspond to CC genotype, 244, 172 and 72 bp represent the heterozygous CT genotype and 244 bp represents TT genotype. For MDR1 G2977T, single band of 107 bp represent GG genotype, bands of 24, 83, 107 represents GT genotype and bands 24, 83 bp represent TT genotype. However, 24 bp band cannot be visualized, this makes GT presented with 83 and 107 bp and TT presented with 83 alone. This is illustrated in Fig. 2.

### Statistical analysis

The SPSS software (Statistical Package for the Social Sciences, version 25, SPSS Inc, Chicago, Ill, USA) was used for analysis of data. Quantitative data were presented in mean and Standard Deviation (SD) or median and interquartile range (IQR). Qualitative data were presented as number (N) and percent (%). P value ≤ 0.05 was considered to be statistically significant. To compare between 2 studied groups, **Student T** test was used for parametric quantitative variables and **Mann Whitney Test (U test)** was used to for non-parametric variables. **The Kruskal-Wallis** test was used to compare none parametric variables, between multiple studied groups. **Chi-Square test** was used to examine the relationship between two qualitative variables and **Fisher’s exact test** was used to examine the relationship between two qualitative variables when the expected count is less than 5 in more than 20% of cells. Odds ratio (OR) and 95% confidence interval (CI) were calculated to estimate the strength of the associations. The genes variants under investigation were evaluated for deviation from Hardy–Weinberg equilibrium (HWE) by comparing observed and expected genotype frequencies in control groups. **Kaplan–Meier test** was used for survival analysis and the statistical significance of differences among curves was determined by Log-Rank test.

## Results

The study overall included 163 subjects; 83 pediatric KTRs and 80 age / sex matched healthy controls **(p = 0.06 and 0.6 receptively).** The mean age of KTRs at time of KT was 9.3 ± 2.9 years, with a median post-transplant follow up duration of 5 years and male to female ratio was 3.6. CAKUT represented 50.6% of original kidney disease. Pre-emptive KT was performed in 4 patients (4.8%), while 95.2% of cases required hemodialysis prior to transplantation. Preemptive plasma exchange (PE) was indicated in 15 patients (18.1%) due to sporadic FSGS as center policy [35]. Demographic, clinical characteristics and transplantation related data of included patients are summarized in Table 1.

**Table 1:** Clinical characteristics & KT related data of included KTRs (n = 83)

Age at transplantation (years)		Mean ± SD	9.3 ± 2.9
Post-transplant follow-up duration (months)		median (IQR)	60 (36–93)
Original kidney disease	CAKUT	N (%)	42 (50.6%)
	NONE CAKUT	N (%)	41 (49.4%)
Family history of any kidney disease		N (%)	6 (7.2%)
Patients required hemodialysis before transplantation		N (%)	79 (95.2%)
Pre-epmptive kidney transplantation		N (%)	4 (4.8%)
Hemodialysis duration (months)		median (IQR)	12 (1–60)
Native nephrectomy	No	N (%)	43 (51.8%)
	Unilateral	N (%)	12 (14.5%)
	Bilateral	N (%)	28 (33.7%)
Cause of nephrectomy	Heavy proteinuria	N (%)	17 (42.5%)
	Large polycystic kidney	N (%)	2 (5%)
	Pyelonephritic kidney	N (%)	6 (15%)
	Marked hydronephrosis	N (%)	15 (37.5%)
Anthropometric measures at enrollment in the study	Weight (Kg)	Mean ± SD	37 ± 11.9
	Height (m)	Mean ± SD	1.36 ± 0.17
	BMI (kg/m^2^)	Mean ± SD	19.4 ± 4.5
*CMV risk stratification	Low risk	N (%)	8 (9.6%)
	Intermediate	N (%)	70 (84.3%)
	High risk	N (%)	5 (6%)
Donor/recipient HLA mismatch	4/6	N (%)	3 (3.6%)
	3/6	N (%)	50 (60.2%)
	2/6	N (%)	25 (30.1%)
	1/6	N (%)	3 (3.6%)
	0/6	N (%)	2 (2.4%)
Antibody induction therapy	Basiliximab	N (%)	43 (51.8%)
	ATG	N (%)	40 (48.2%)
Preemptive PEX		N (%)	15 (18.1%)
Maintenance IS other than TAC	MMF	N (%)	82 (98.8%)
	Azathioprine	N (%)	1 (1.2%)

KT; kidney transplantation, KTR ; kidney transplant recipients, N; number, SD; standard deviation, IQR; interquartile range, CAKUT; congenital anomalies of kidney and urinary tract, Kg; kilograms, m; meter, BMI; body mass index, CMV; cytomegalo virus, HLA; human leukocytic antigen, ATG: anti thymocyte globulin, PEX; plasma exchange, IS; immunosuppressive, TAC; tacrolimus, MMF; mycophenolic mofetil.* CMV IgG: Low risk (D -, R -), intermediate risk either (D+, R+) or (D-, R+), high risk (D+, R-)

Patients with AR (n = 36) were diagnosed pathologically as ABMR in 38.9%, TCMR in 50% and Mixed rejection in 11.1% of patients. The median duration of post-transplant follow-up duration was 60 months with interquartile range (IQR) (36–93 months). The onset of AR was early (in 1st 3 months post-transplant) in 9 cases and delayed (between 3 and 14 months post-transplant) in the remaining 27 cases. Median TAC trough level at time of AR was 5 with median TAC dose of 0.14 mg/kg/day. Pulse methyl prednisone was the first line antirejection therapy received by all AR cases with further therapy depending on the pathological type of AR. Patients with ABMR received PEX, rituximab (RTX) and IVIG while ATG received mainly by patients with TCMR. Fortunately, 52.8% of cases had complete response, 38.9% achieved partial response, and only 8.3% did not achieve any response. Complete response was defined as return of serum creatinine after treatment to 25% or less above the basal creatinine, partial remission was considered if creatinine remained 25–75% above basal level and no response if none of the above mentioned definitions was fulfilled [36].

The MDR1, C3435T and G2677T genotypes and alleles frequencies were compared between AR group and 2 control groups; healthy controls and disease control (none AR cases) (Table 2). The frequency of the CC, CT genotypes and C allele were significantly higher among AR KTRs than their frequency among None AR KTRs **(p = 0.008, 0.001 and 0.01 respectively).** Moreover; AR group had more frequent CC, CT genotypes and C allele than healthy controls **(p = 0.006, 0.028 and 0.008 respectively)**. However, no significant difference was observed in the frequency of either genotypes or alleles between the None AR group and healthy controls **(p > 0.05)**.

**Table 2:** Frequency of C3435T and G2677T genotypes and allelic polymorphisms in KTRs (AR, non-AR groups) and controls

			All KTRs (n = 83)	AR group (n = 36)	None AR group (n = 47)	Controls (n = 80)	AR vs. None AR		AR vs. Controls		None AR vs. Controls	
			N (%)	N (%)	N (%)	N (%)	OR (95% CI)	P value	OR (95% CI)	P value	OR (95% CI)	P value
**C3435T**	**Genotype**	**TT**	22 (26.5)	3 (8.3)	19 (40.4)	24 (30)	**Reference**	-	**Reference**	-	**Reference**	-
		**CT**	35 (42.2)	20 (55.6)	15 (31.9)	40 (50)	3.58 (1.64–7.80)	**0.001**	2.20 (1.08–4.46)	**0.028**	0.63 (0.37–1.06)	0.082
		**CC**	26 (31.3)	13 (36.1)	13 (27.7)	16 (20)	2.99 (1.32–6.75)	**0.008**	2.98 (91.37–6.46)	**0.006**	1.02 (0.56–1.83)	0.957
	**Allele**	**T**	79 (47.6)	26 (36.1)	53 (56.4)	88 (55)	**Reference**	**-**	**Reference**	**-**	**Reference**	-
		**C**	87 (52.4)	46 (63.9)	41 (43.6)	72(45)	1.67 (1.13–2.46)	**0.010**	1.59 (1.13–2.24)	**0.008**	0.97 (0.70–1.32)	0.83
**G2677T**	**Genotype**	**GG**	23 (27.7	5 (13.9)	18 (38.3)	39 (48.8)	**Reference**	-	**Reference**	-	**Reference**	-
		**GT**	37 (44.6	19 (52.8)	18 (38.3)	28 (35)	2.259 (1.12–4.555)	**0.023**	2.625 (1.43–4.817)	**0.002**	1.226 (0.743–2.021)	0.425
		**TT**	23 (27.7)	12 (33.3)	11 (23.4)	13 (16.3)	2.306 (1.069–4.975)	**0.033**	3.181 (1.592–6.356)	**0.001**	1.455 (0.793–2.668)	0.226
	**Allele**	**G**	83 (50)	29 (40.3)	54 (57.4)	106 (66.3)	**Reference**	-	**Reference**	-	**Reference**	-
		**T**	83 (50)	43 (59.7)	40 (42.6)	54 (33.8)	1.541 (1.047–2.268)	**0.028**	1.91 (1.353–2.697)	**< 0.001**	1.261 (0.911–1.744)	0.162

KTR; kidney transplant recipients, N; number, OR; odds ratio, CI; confidence interval, C; cytosine, T; thymine, G; guanine. Logistic regression analysis was used. AR; acute rejection; N; number, OR; odds ratio, CI; confidence interval, C; cytosine, T; thymine. Logistic regression analysis was used. Reference genotype and allele according to NCBI. P < 0.05 is considered significant; OR < 1 is considered protective; OR > 1 is considered risky

While in G2677T, the frequency of GT, TT genotypes and T allele were significantly higher among AR KTRs than their frequency among None AR KTRs **(p = 0.023, 0.033 and 0.028 respectively).** In addition, the AR group had more frequent GT, TT genotypes and T allele than healthy controls **(p = 0.002, 0.001 and < 0.001 respectively)**.

The effect of combined genotypes is illustrated in Table 3, both CT _3435_GT _2677_ and CT _3435_ TT _2677_ were significantly higher in AR KTRs than none AR **(P value = 0.024 and 0.013 respectively).**

**Table 3:** Association of combined genotypes (C3435T and G2677T) with AR in AR and none AR cases

Variable	AR (n = 36)	None AR (n = 47)	OR (95% CI)	P value
	N (%)	N (%)		
**TT**_**3435**_ **GG**_**2677**_	3 (8.3%)	10 (21.3%)	**Reference**	
**CC**_**3435**_ **GT**_**2677**_	8 (22.2%)	6 (12.8%)	2.500 (0.918–6.806)	0.073
**CC**_**3435**_ **TT**_**2677**_	5 (13.9%)	6 (12.8%)	1.863 (0.647–5.363)	0.249
**CT**_**3435**_ **GG**_**2677**_	2 (5.6%)	7 (14.9%)	0.972 (0.298–3.172)	0.962
**CT**_**3435**_ **GT**_**2677**_	11 (30.6%)	6 (12.8%)	3.046 (1.155–8.033)	**0.024**
**CT**_**3435**_ **TT**_**2677**_	7 (19.4%)	2 (4.3%)	4.486 (1.375–14.639)	**0.013**
**CC**_**3435**_ **GG**_**2677**_	0 (0%)	1 (2.1%)	-	1
**TT**_**3435**_ **GT**_**2677**_	0 (0%)	6 (12.8%)	-	1
**TT**_**3435**_ **TT**_**2677**_	0 (0%)	3 (6.4%)	-	1

AR; acute rejection; N; number, OR; odds ratio, CI; confidence interval, C; cytosine, T; thymine, G; guanine. Logistic regression analysis was used. P < 0.05 is considered significant; OR < 1 is considered protective; OR > 1 is considered risky. Reference combined genotype was considered as TT _3435_ GG _2677_

The trough levels of TAC, required doses and C/D ratio during the first six months after KT were compared between different genotypes of both SNPs as in Table 4. As regard C3435T, the trough levels were lower among CC than CT than TT genotypes, however it did not reach statistically significant p value. In addition, the required TAC dose needed to achieve the target trough level was significantly higher among CC than CT than TT genotypes through the 1st 6 months posttransplant **(p < 0.001).** Consequently, the C/D ratio decreased significantly in cases carrying CC then CT then TT genotypes through the 1st 6 months post- transplant **(p < 0.001).** Similarly, in G2677T, the trough levels were lower among TT than GT than GG genotypes, that reached statistically significant p value in 3rd month and the mean trough level of 1st six months. In addition, the required TAC dose needed to achieve the target trough level was significantly higher among TT than GT than GG genotypes through the 1st 6 months posttransplant **(p < 0.001).** Consequently, the C/D ratio decreased significantly in cases carrying TT than GT than GG genotypes through the 1st 6 months post- transplant **(p < 0.001).**

**Table 4:** Comparison of tacrolimus doses and trough levels according to the C3435T and G2677T genotypes

	C3435T genotypes				G2677T genotypes			
Variables	CC (n = 26)	CT (n = 35)	TT (n = 22)	P value	GG (n = 23)	GT (n = 37)	TT (n = 23)	P value
	Median (IQR)				Median (IQR)			
**Trough level (ng/ml)**								
**1st month**	7.9 (5.8-9)	7.3 (6-9.5)	7.8 (7-9.3)	0.9	7.6 (4.3–9.9)	7.9 (2.5-8)	7.6 (2.5–8.3)	0.896
**2nd month**	8 (6.3–9.4)	9.8 (7.2–10)	9.8 (7–11)	0.7	9.9 (5.2–10.3)	9.5 (5.2–10.3)	9.7 (6–10)	0.530
**3rd month**	8 (6.7–9.6)	8.5 (6.7–10.2)	9.2 (8.5–10.5)	0.083	9.2 (4.5–9.5)	9 (4.5–10)	7.5 (4.5-9)	**0.012**
**4th month**	7.2 (6-9.7)	9.3 (7.2–10.3)	9 (8–10)	0.2	9.3 (4.4–10)	8.3 (4.2–9.6)	7.7 (5.1–8.7)	0.081
**5th month**	7.4 (5–10)	8.4 (5-10.2)	8.2 (5-9.8)	0.2	8.4 (5-9.5)	8.3 (5-9.7)	7 (5-8.4)	0.135
**6th month**	6.6 (6-8.5)	7.4 (4.7–9.6)	7 (4.7–9.1)	0.1	7.4 (4.4-9)	7.2 (2.5-8)	6 (4.5–7.9)	0.850
**Mean trough level of 1st 6 months (ng/ml)**	8 (5.9–9.3)	8.5 (6.1–9.9)	8.65 (6.7–9.95)	0.154	9.18 (4.6–9.9)	8.51 (6.43–9.5)	7.98 (6.21–8.5)	**0.043**
**TAC dose (mg/kg/day)**								
**1st month**	0.3 (0.2–0.3)	0.18 (0.15–0.2)	0.16 (0.14–0.18)	**< 0.001**	0.17 (0.08–0.23)	0.18 (0.1–0.26)	0.27 (0.14–0.3)	**< 0.001**
**2nd month**	0.27 (0.2–0.3)	0.18 (0.15–0.19)	0.18 (0.15–0.19)	**< 0.001**	0.17 (0.04–0.25)	0.18 (0.13–0.26)	0.26 (0.13–0.29)	**< 0.001**
**3rd month**	0.28 (0.2–0.31)	0.18 (0.15–0.2)	0.15 (0.13–0.19)	**< 0.001**	0.15 (0.04–0.23)	0.18 (0.11–0.25)	0.28 (0.13–0.3)	**< 0.001**
**4th month**	0.29 (0.18–0.3)	0.16 (0.13–0.24)	0.12 (0.08–0.16)	**< 0.001**	0.13 (0.04–0.21)	0.16 (0.07–0.2)	0.29 (0.12–0.3)	**< 0.001**
**5th month**	0.24 (0.17–0.3)	0.17 (0.13–0.19)	0.1 (0.06–0.17)	**< 0.001**	0.14 (0.05–0.22)	0.17 (0.06–0.21)	0.23 (0.1–0.26)	**< 0.001**
**6th month**	0.23 (0.18–0.3)	0.17 (0.13–0.23)	0.13 (0.08–0.15)	**0.001**	0.13 (0.07–0.2)	0.18 (0.04–0.22)	0.23 (0.1–0.25)	**0.003**
**Mean TAC dose (mg/kg/day)**	0.27 (0.2–0.32)	0.17 (0.15–0.2)	0.14 (0.12–0.19)	**< 0.001**	0.15 (0.055–0.22)	0.17 (0.115–0.23)	0.27 (0.12–0.28)	**< 0.001**
** C/D ratio (ng/ml/mg/kg/day)**								
**1st month**	25 (17.6–51.2)	49.5 (31.1–64.6)	54.3 (38.1–66.7)	**0.002**	51.18 (23.33–100.5)	40 (8.33–62.86)	35 (8.33–62.5)	**0.024**
**2nd month**	25.5 (23.8–50)	55.6 (43.9–72)	55 (35.6–71.4)	**< 0.001**	64.71 (18.67-80)	50 (20.83–77.22)	25.53 (9.43–79.23)	**0.001**
**3rd month**	26.9 (21.4–37.2)	45 (32.5–90)	65.4 (50.7-105.6)	**< 0.001**	65.38 (29.63-90)	40.63 (14.43–66.3)	26.92 (14.43–57.4)	**< 0.001**
**4th month**	27.1 (20.7–42.5)	49 (38-66.3)	72.7 (54.7–150)	**< 0.001**	66.25 (20.38–70.5)	42.86 (8.95–67.5)	27.08 (8.95–47.67)	**< 0.001**
**5th month**	30.4 (21.8–41)	41.2 (33.3–78.8)	69.2 (57.9-101.7)	**< 0.001**	60 (28-96.67)	41.18 (10.38–76.6)	25.16 (10.38–65.4)	**< 0.001**
**6th month**	30 (21.4–42.9)	43 (30.7–58.8)	58.8 (38.3–91)	**0.001**	56.3 (28.7–86.9)	42.9 (13.8–77.5)	26.7 (13.1–60.6)	**0.002**
**Mean C/D ratio in 1st six months**	30.2 (22.4–38.5)	47.3 (38.5–74)	68.1 (49.9-106.3)	**< 0.001**	68.1 (26.6–90)	47.3 (13–83)	29.2 (13-75.2)	**< 0.001**

N; number, IQR; inter quartile range, TAC; tacrolimus, C; cytosine, T; thymine, G; guanine, ng/ml; nanogram per milliliter, C/D; trough concentration/dose, Kruskal Wallis test was used for comparison

No significant difference was detected between different MDR1 C3435T and G2677T genotypes as regard frequency of TAC related adverse effects (Table 5).

**Table 5:** Comparison between C3435T and G2677T genotypes regarding tacrolimus side effects

Variables	N (%)			
C3435T	CC (N = 26)	CT (N = 35)	TT (N = 22)	P value
**Nephrotoxicity**	5 (19.2%)	10 (28.6%)	5 (22.7%)	0.690
**Neurotoxicity**	1 (3.8%)	1 (2.9%)	5 (22.7%)	0.670
**NODAT**	0 (0%)	1 (2.9%)	0 (0%)	0.500
**Dyslipidemia**	1 (3.8%)	2 (5.7%)	1 (4.5%)	0.943
**hirsutism**	0 (0%)	1 (2.9%)	0 (0%)	0.500
**G2677T**	**GG (N = 19)**	**GT (N = 44)**	**TT (N = 20)**	**P value**
**Nephrotoxicity**	9 (39.1%)	8 (21.6%)	3 (13.0%)	0.105
**Neurotoxicity**	0 (0%)	1 (2.7%)	1 (4.3%)	0.622
**NODAT**	1 (4.3%)	0 (0%)	0 (0%)	0.554
**Dyslipidemia**	1 (4.3%)	2 (5.4%)	1 (4.3%)	0.975
**hirsutism**	1 (4.3%)	0 (0%)	0 (0%)	0.554

TAC; tacrolimus, N; number, C; cytosine, T; thymine, G; guanine, NODAT; new onset diabetes after transplantation, Chi square test was used for comparison

Regression analysis was conducted for prediction of AR, using many covariates as recipient age, gender, original kidney disease, need for dialysis and its duration prior to transplantation, CMV risk stratification, degree of HLA mismatch, type of induction therapy, need for pre-emptive PEX and the maintenance IS therapy. None of the above-mentioned factors was associated with risk of AR as in Table 6.

**Table 6:** Analysis of clinical, immunological characteristics and immunosuppressive therapy as predictors for AR in AR versus none AR cases

Variables			AR group N = 36	Non-AR N = 47	OR (95% CI)	*P* *Value*
**Age at transplant**		**Mean ± SD**	9.4 (3.2%)	10 **±** 2.8	0.953(0.819–1.109)	0.535
**Sex**	**Male**	**N (%)**	30 (83.3%)	35 (74.5%)	**Reference**	-
	**Female**	**N (%)**	6 (16.7%)	12 (25.5%)	0.552(0.183–1.66)	0.290
**Original kidney disease**	**NONE CAKUT**	**N (%)**	17 (47.2%)	24 (51.1%)	**Reference**	-
	**CAKUT**	**N (%)**	19 (52.8%)	23 (48.9%)	1.101 (0.640–1.893)	0.744
**Dialysis before Tx**	**No**	**N (%)**	2 (5.6%)	2 (4.3%)	**Reference**	-
	**Yes**	**N (%)**	34 (94.4%)	45 (95.7%)	1.324(0.177–9.877)	0.785
**Dialysis duration (months)**		**Median, IQR**	12 (2–60)	12 (1–60)	0.979(0.936–1.025)	0.366
**Donor/recipient HLA mismatch**	**4/6**	**N (%)**	0	3 (6.4)	**Reference**	-
	**3/6**	**N (%)**	22 (61.1)	28 (59.6)	-	0.1
	**2/6**	**N (%)**	12 (33.3)	13 (27.7)	-	0.1
	**1/6**	**N (%)**	2 (5.6)	1 (2.1)	-	0.1
	**0/6**	**N (%)**	0	2 (4.3)	-	-
CMV risk stratification*	**Low risk**	**N (%)**	5 (13.9%)	3 (6.4%)	**Reference**	-
	**Intermediate**	**N (%)**	30 (83.3%)	40 (85.1%)	0.607(0.239–1.543)	0.295
	**High risk**	**N (%)**	1 (2.8%)	4 (8.5%)	0.313(0.068–1.452)	0.138
**Antibody induction**	**Basiliximab**	**N (%)**	15 (41.7%)	28 (59.6%)	**Reference**	-
	**ATG**	**N (%)**	21 (58.3%)	19 (40.4%)	1.57(0.909–2.714)	0.106
**Preemptive PEX**	**NO**	**N (%)**	32 (88.9%)	36 (76.6%)	**Reference**	-
	**Yes**	**N (%)**	4 (11.1%)	11 (23.4%)	0.577(0.275–1.215)	0.148
**Maintenance IS other than TAC**	**MMF**	**N (%)**	36 (100%)	46 (97.9%)	-	0.379
	**Azathioprine**	**N (%)**	0 (0%)	1 (2.1%)		

AR; acute rejection, N; number, SD; standard deviation, IQR; interquartile range, CAKUT; congenital anomalies of kidney and urinary tract, CMV; cytomegalo virus, HLA; human leukocytic antigen, ATG: anti thymocyte globulin, PEX; plasma exchange, IS; immunosuppressive, TAC; tacrolimus, MMF; mycophenolic mofetil.* CMV IgG: Low risk (D -, R -), intermediate risk either (D+, R+) or (D-, R+), high risk (D+, R-), Logistic regression analysis test was used for statistical analysis

**Fig. 3:**
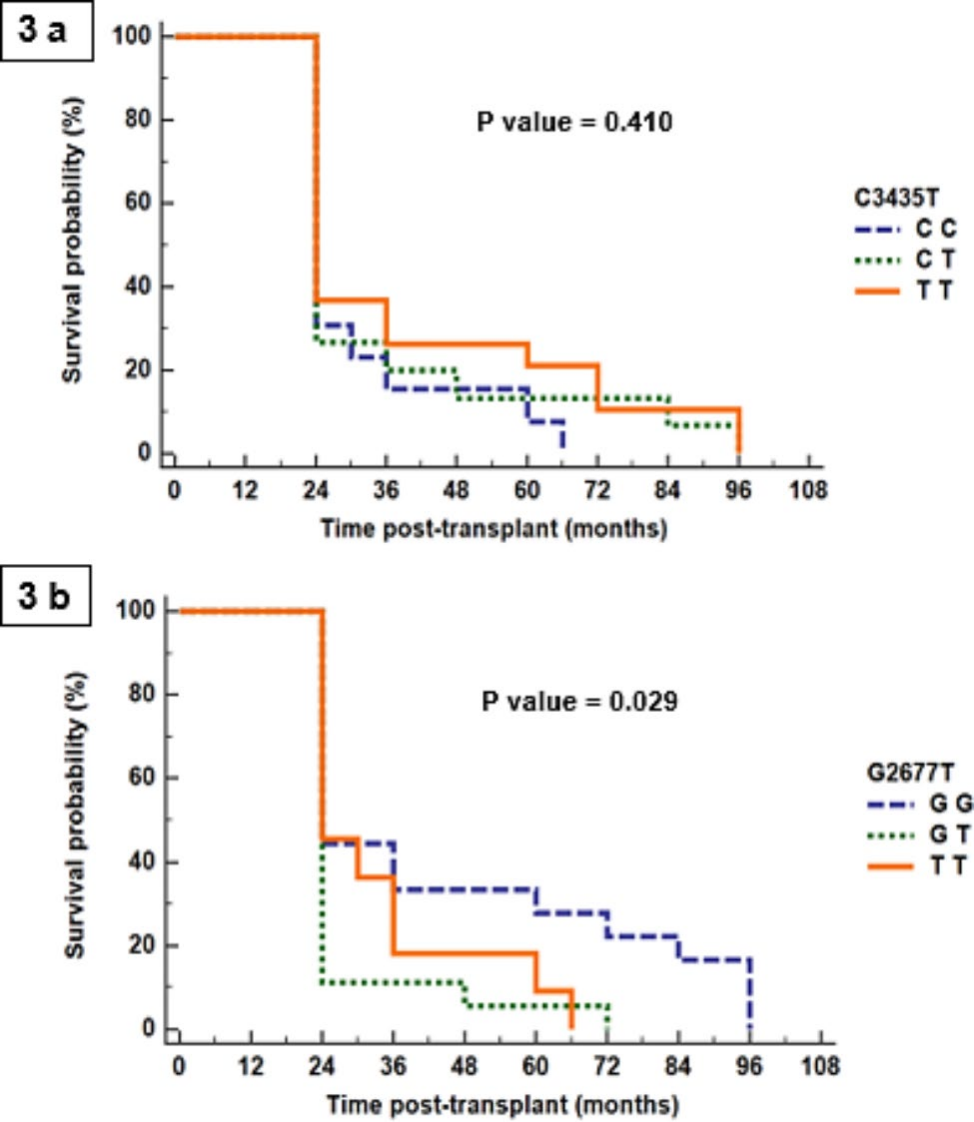
Survival analysis of AR cases, **3a** represents C3435T and **3b** represents G2677T genotype

Kaplan-Meier analysis was conducted for time lapse after kidney transplantation in AR group. Cumulative survival proportions as well as median survival time are shown in Fig. 3. As regard C345T polymorphism, no significant difference was found between genotypes **(p = 0.410).** While in G2677T polymorphism, significantly longer survival was found in GG genotype than TT and GT genotype **(p = 0.029).**

## Discussion

To the best of our knowledge, this is the first study that analyses the role of MDR1 gene polymorphism in KT among children in Egypt and Arab countries. It is important to draw up a different treatment plan for each KT recipient. As MDR1 shows great heterogeneity among different ethnic groups, there is a need for pharmacogenomic testing prior to TAC administration to achieve genotype-guided dose and contribute to a better-individualized IS therapy. In the present study, we evaluated the impact of MDR1, C3435T and G2677T on the occurrence of AR and variability in the TAC pharmacokinetics in pediatric KTRs.

Considering TT genotype as a reference genotype and T allele as a reference allele in C3435T SNP; the current study revealed that the CC, CT genotypes and C allele were significantly associated with risk of AR when compared to none AR and to the healthy controls. This means that the presence of single C allele of C3435T can be a risk factor of AR in KTRs. When comparing none AR versus Control, no significant difference was found regarding C3435T genotypes and alleles with none AR **(p > 0.05 for each).**

Our results are quite similar to another pediatric study of KTRs that reported higher incidence of AR in CT genotype, without reaching the statistically significant value. It was suggested by the authors that the presence of wild type, C allele increases drug efflux out of cells and decreases drug concentrations in the target cells which eventually leads to AR. It is difficult to explain why this effect was not observed in the homozygous CC genotype. However, it can be attributed to small number of cases (only 38 cases from Saudi Arabia) in their study [37]. Our results also are in concordance with Zheng’s study in American population that reported lower incidence of AR among those with TT genotype [38].

In G2677T, considering GG genotype and G allele as references, both GT, TT genotypes and T allele were found to be significantly higher among AR cases that means that T allele is considered a risk for AR in KTRs. This may be further confirmed by the survival analysis that revealed best survival with GG (none T allele containing) genotype.

The current findings are similar to another study in Caucasian population with higher incidence of AR in TT and GT than GG genotype [39]. However, our results are contradictory to the conclusion of an adult Egyptian study that G2677T/A did not differ between rejecters and non-rejecters. This may be attributed to limited number of cases in their study (only 50 cases) and rejection only in 2 cases. In addition, their cohort received cyclosporin rather than TAC based triple IS maintenance therapy [40].

The effect of combined genotypes indicates that both CT _3435_GT _2677_ and CT _3435_ TT _2677_ were significantly higher in AR KTRs than none AR. This finding can be explained by linkage disequilibrium (LD) between both SNPs that may contribute to functional alteration rather than the effect of single haplotype variation [23].

In the present study; assessment of patients′ TAC trough levels and required doses, across 1st 6 months posttransplant, revealed that higher TAC doses and lower C/D ratio were observed among CC than CT than TT genotypes of C3435T, through the 1st 6 months. The current results mean that patients with CC genotypes had difficulty to achieve the trough levels and required higher doses to reach it in comparison to CT and TT genotypes. This effect can be explained by the role of C3435T SNP, in regulating the expression of P-gp expression and controlling efflux of TAC, other drugs and toxic metabolites [15]. It was proven that, individuals with wild CC genotype had much higher expression of P-gp (efflux transporter that excrete drugs and toxic substances outside the cells) [14].

The current results are similar to the data reported in both kidney and liver transplantation [9, 41]. Similarly, another study from Egypt, about effect of C3435T on dose of TAC and C/D ratio in liver Tx, found that higher doses and lower C/D ratios were observed in the wild CC genotype of the graft (the donor genotype). The condition in liver Tx is quite different from KT, as liver is the 1ry site of metabolism of most of IS drugs. This effect was most evident 6 months post Tx as the liver graft become fully functioning and was independent from the recipient genotypes [42].

The current study found that TT genotype of G2677T required higher TAC doses to achieve the target trough level than the GT and GG genotypes, with lower C/D ratio. Our results are contradictory to Mai et al. study. In the latter study; the authors concluded that both C3435T and G2677T SNPs do not affect TAC pharmacokinetics in KT recipients with stable graft function [43]. In addition, another study concluded that neither C3435T nor G2677T SNPs contributed to variability in TAC dose requirement or AR episodes in KT [44]. This difference can be attributed to ethnic variability, small sample size, the combined effect of other genotypes and enzymes involved in the metabolism of IS drugs.

Tacrolimus is not only a substrate of P-gp, but also it undergoes extensive metabolism by intestinal and hepatic CYP3A4 and CYP 3A5. Thus, TAC intestinal efflux mediated by P-gp is not the only mechanism altering the drug bioavailability. It was actually concluded from another study that *CYP3A5* genotype can affect TAC dose requirements [45]. In addition, MDR1 also can affect steroid efflux and pharmacokinetics in many diseases as nephrotic syndrome and ulcerative colitis that can explain the effect of different genotypes and alleles on the incidence of AR (in spite of achieving the TAC trough level) [46].

As regard the effect of MDR1 SNP genotypes and haplotypes on TAC induced adverse effects, including nephrotoxicity, neurotoxicity, new onset diabetes after transplantation (NODAT), dyslipidemia and hirsutism, no significant association was found between both C3435T and G2677T genotypes and TAC side effects. This is similar to another study that found no significant association between another MDR1 gene SNP (G2677T) and the TAC related nephrotoxicity, neurotoxicity or hypertension [47]. On the other hand, another study concluded that variation in both genotypes and haplotypes of C3435T and G2677T SNPs may play a role in occurrence of TAC adverse effects in liver Tx [48]. Further studies are essential to prove or exclude this association to provide adequate tools to predict the drug effects and toxicity.

The regression analysis of many risk factors for AR as the degree of HLA mismatch, CMV risk and utilized induction and maintenance therapy revealed that no significant difference was found between the AR and none AR groups. This finding supports our argument that MDR 1 genotypes and alleles polymorphism has an impact on occurrence of AR. This can be explained by effect of MDR1 pharmacogenetics on tacrolimus metabolism. Although all of our cohort achieved the trough levels, the risky genotypes (CC genotype of C3435T and TT genotype of G2677T) had relatively lower levels than other genotypes. In addition, they required higher doses and frequent titration of the dose to achieve the trough levels so they were more predisposed to the risk of AR.

As regard the similarity between our control cohort and other documented genotypes of both studied SNPs in the different populations, published in the literature, no significant difference was found and no deviation from Hardy-Weinberg equilibrium (HWE) was observed (Table 7). Study form Saudi Arabia was excluded as genotypes were not in Hardy Weinberg equilibrium. Genetic similarities were found with all other populations in different studies. The points of strength of the current study include being in a peculiar age group of Egyptian population and relatively adequate number of patients. However, it is limited by being a single center study.

**Table 7:** Comparison of genetic variability in MDR 1 C3435T and G2677T between Egyptian health controls with controls from other studies

	MDR1 C3435T								
First author/ Reference	Country	Control number	CC	CT	TT	C	T	HW p	F_ST_ vs. current
**Korkor et al.; [current]**	**Egypt**	80	16	40	24	0.450	0.550	0.928	-
[49].	**Jordan**	116	24	60	32	0.466	0.534	0.671	< 0.001
[49].	**Sudan**	131	69	55	7	0.737	0.263	0.348	0.045
[50].	**Morocco**	100	39	51	10	0.645	0.355	0.256	0.038
[51].	**Bahrain**	184	64	84	36	0.576	0.424	0.376	0.016
[52].	**Iran**	200	40	105	55	0.463	0.538	0.429	< 0.001
[53].	**Turkey**	150	30	80	40	0.467	0.533	0.382	< 0.001
[54].	**Egypt**	200	68	103	29	0.598	0.403	0.317	0.022
[8].	**France**	81	29	34	18	0.568	0.432	0.193	0.014
[14].	**Germany**	188	53	90	45	0.521	0.479	0.576	0.005
	**MDR1 G2677T**								
**First author/ Reference**	**Country**	**Control number**	**GG**	**GT**	**TT**	**G**	**T**	**HW p**	**F**_**ST**_vs. **current**
**Korkor et al.; [current]**	**Egypt**	80	39	28	13	0.663	0.338	0.052	-
[33].	**Iran**	120	78	25	0	0.879	0.121	0.161	0.066
[55].	**China**	200	35	75	42	0.477	0.523	0.892	0.035
[56].	**Brazil**	106	42	42	19	0.612	0.388	0.151	0.003
[57].	**England**	285	94	135	42	0.596	0.404	0.571	0.005
[58].	**Korea**	632	120	214	103	0.519	0.481	0.690	0.021
[59].	**Scotland**	370	102	174	94	0.511	0.489	0.256	0.024

P value (P value number significant if ≤ 0.05). MRD1: multi drug resistant type 1, T: thymine, G: guanine, C; cytosine, Genotypes are expressed as number, alleles are expressed as frequencies; HW p, p value of Hardy Weinberg equation; Fst: Comparisons were done using pairwise fixation index (FST) comparison versus the current study

## Conclusion

MDR1, C3435T and G2677T SNPs may have a role in tailoring immunosuppressive regimen in pediatric KTRs. CC, CT genotypes and C allele of C3435T and GT, TT genotypes and T allele of G2677T SNP, could carry a risk for AR due to difficulty in achievement of TAC trough level. To date, there is no consensus on routine MDR 1 genotyping prior to solid organ transplantation to guide the choice of appropriate TAC dose. However, analysis should be performed in resourceful settings to guide TAC dose and anticipating potential outcomes as AR or drug toxicity.

## Electronic supplementary material

Below is the link to the electronic supplementary material.

Supplementary Material 1

## Data Availability

The datasets used and/or analysed during the current study are available from the corresponding author on reasonable request.

## List of Abbreviations

AR: Acute rejection
AMR: Antibody mediated rejection; globulin
Bp: Base pair
CI: Confidence interval
CKD: Chronic kidney disease
C/D: Concentration/dose
CAKUT: Congenital anomalies of the kidney and the urinary tract
C: Cytosine
CMV: Cytomegalo virus
ESKD: End stage kidney disease
EDTA: Ethylene-diamine-tetra-acetic acid
Fst: Fixation index
FSGS: Focal segmental glomerulosclerosis
GWASs: Genome wide association studies
G: Guanine
HWE: Hardy–Weinberg equilibrium
HLA: Human leukocytic antigen
IVIG: Intra venous immunoglobulins
HIV: Human immunodeficiency virus
IS: Immunosuppressive
KT: Kidney transplantation
KTR: Kidney transplant recipients
LN: Lupus nephritis
mTORi: Mammalian target of rapamycin inhibitor
MCDK: Multicystic dysplastic kidneys
MDR-1: Multidrug resistant 1
MPGN: Membranoproliferative glomerulonephritis
MMF: Mycophenolate mofetil
N: Number
OR: Odds ratio
P: P value
P-gp: Permeation glycoprotein
PEX: Plasma exchange
PCR-RFLP: Polymerase chain reaction-restriction fragment length polymorphism
PUJO: Pelvi-ureteric junction obstruction
PUV: Posterior urethral valve
QOL: Quality of life
ATG: Anti-thymocyte globulin
RTX: Rituximab
SNP: Single nucleotide polymorphism
SD: Standard deviation
SPSS: Statistical Package for the Social Sciences
TCMR: T cell mediated rejection
TAC: Tacrolimus
T: Thymine
TG: Transplant glomerulopathy
Vs: Versus
VUR: Vesicoureteral reflux
