# Supplementary material for: Multidrug resistant 1 (MDR1) C3435T and G2677T gene polymorphism: impact on the risk of acute rejection in pediatric kidney transplant recipients
Source: Ital J Pediatr. 2023 May 18;49:57. doi: 10.1186/s13052-023-01469-w (PMC10193607; doi:10.1186/s13052-023-01469-w)
Supplement: Supplementary file 1 — Supplementary Material 1 [file 13052_2023_1469_MOESM1_ESM.docx]

**Response to reviewer’s Comments**

**To editor:** Thanks a lot for considering our work for evaluation and possible publication in “Italian journal of pediatrics”. Please find below response to reviewer’s comments point by point and the required changes were done to the manuscript tracked and highlighted.

**To reviewers:** Thanks a lot for your careful revision of our work and valuable comments. We have tried to address all the comments as much as possible. Please find response bellow and the required modifications were done to the manuscript.

**Reviewer 1:**

| **Comment** | **Response to reviewer comment** | **Changes made within the revised manuscript** |
| --- | --- | --- |
| **Reviewer #1:**  I appreciate that the authors have addressed the points I have made.  The content of this manuscript has become clearer and better understood.  The authors have made almost complete corrections, but one point should be added to the p-value of the following sentence;  Page 15, L40-  Kaplan-Meier analysis was conducted for time lapse after kidney transplantation in  AR group. Cumulative survival proportions as well as median survival time are shown in  figure 3. As regard C345T polymorphism, no significant difference was found between  genotypes (p=0.410). While in G2677T polymorphism, significantly longer survival was found in GG genotype than TT and GT genotype (p=0.029). | - The p-value of the survival analysis of AR cases regarding C3435T SNP was added as requested. | - The requested p-value was added in last paragraph of "results" section of the manuscript (written in bold and highlighted in green). |

**Reviewer 2:**

| **Comment** | **Response to reviewer comment** | **Changes made within the revised manuscript** |
| --- | --- | --- |
| **Reviewer #2:** I have just one minor comment:  Page 9 There was a text added describing CAKUT and non CAKUT patients during revision, however, the subsequent sentence does not make sense. | - The subsequent sentence actually does not make sense. I tried to rewrite it in a more appropriate manner. | - The added words required to make a meaningful sentence were added in the 5^th^ paragraph in "patients and methods" section (written in red). |
